# Using *Wolbachia* to Eliminate Dengue: Will the Virus Fight Back?

**DOI:** 10.1128/JVI.02203-20

**Published:** 2021-06-10

**Authors:** Kathryn M. Edenborough, Heather A. Flores, Cameron P. Simmons, Johanna E. Fraser

**Affiliations:** a Institute of Vector-Borne Disease, Monash University, Clayton, Victoria, Australia; b World Mosquito Program, Institute of Vector-Borne Disease, Monash University, Clayton, Victoria, Australia; c Oxford University Clinical Research Unit, Ho Chi Minh City, Vietnam; d Nuffield Department of Medicine, University of Oxford, Oxford, United Kingdom; e Department of Microbiology, Monash Biomedicine Discovery Institute, Monash University, Clayton, Victoria, Australia; National Institute of Allergy and Infectious Diseases

**Keywords:** *Aedes aegypti*, *Wolbachia*, arbovirus, dengue virus, mechanisms of resistance

## Abstract

Recent field trials have demonstrated that dengue incidence can be substantially reduced by introgressing strains of the endosymbiotic bacterium *Wolbachia* into Aedes aegypti mosquito populations. This strategy relies on *Wolbachia* reducing the susceptibility of *Ae. aegypti* to disseminated infection by positive-sense RNA viruses like dengue. However, RNA viruses are well known to adapt to antiviral pressures. Here, we review the viral infection stages where selection for *Wolbachia*-resistant virus variants could occur. We also consider the genetic constraints imposed on viruses that alternate between vertebrate and invertebrate hosts, and the likely selection pressures to which dengue virus might adapt in order to be effectively transmitted by *Ae. aegypti* that carry *Wolbachia*. While there are hurdles to dengue viruses developing resistance to *Wolbachia*, we suggest that long-term surveillance for resistant viruses should be an integral component of *Wolbachia*-introgression biocontrol programs.

## INTRODUCTION

## REDUCING DENGUE INCIDENCE WITH *WOLBACHIA*-BASED BIOCONTROL MEASURES

Every year an estimated 390 million people become infected with dengue virus (DENV; *Flaviviridae*, *Flavivirus*) and the incidence of dengue disease is rising (1, 2). DENV is primarily spread by female Aedes aegypti mosquitoes, which thrive in urban habitats at subtropical and tropical latitudes (3). In the absence of broadly effective therapeutics or vaccines (4–8), disease control efforts have historically involved suppression of mosquito populations by removal of urban breeding habitats and insecticide/larvicide treatment (9). However, the accumulation of insecticide resistance in *Ae. aegypti* populations (10, 11) and continual dengue epidemics have shown these approaches have not been effective. This has driven the innovation and implementation of a range of mosquito “rear and release” methods (12–15), the most advanced of which uses *Ae. aegypti* artificially infected with the endosymbiotic bacterium Wolbachia pipientis (16–20).

The *Wolbachia* introgression approach involves time-limited field release of *Ae. aegypti* infected with *Wolbachia* strains *w*Mel or *w*AlbB (derived from Drosophila melanogaster and Aedes albopictus, respectively). Over time, *Wolbachia* introgresses into the local *Ae. aegypti* population. The result is an *Ae. aegypti* population with a high prevalence of *Wolbachia* infection. Introgression is driven by maternal transmission of *Wolbachia* and a reproductive advantage that the bacterium gives to *Wolbachia*-carrying females, termed cytoplasmic incompatibility (21). Additionally, *Ae. aegypti* infected with *w*Mel or *w*AlbB are less susceptible to disseminated infection with all 4 serotypes of DENV, and are less likely to have infectious virus in their saliva (22, 23). Importantly, epidemiological studies report a substantial and significant reduction in dengue incidence in communities where *w*Mel- or *w*AlbB-mosquitoes have been established (17, 19, 24–26).

## IMPACTS OF EVOLUTION ON *WOLBACHIA* AS A BIOCONTROL TOOL

The ability of *Wolbachia* to provide long term protection against DENV could be undermined by genome evolution of *w*Mel, *Ae. aegypti*, and/or DENV. Evolution of *w*Mel tracks slower than the mitochondrial genome of its natural host, D. melanogaster (27, 28), and sequencing of *w*Mel from *Ae. aegypti* collected 2 to 8 years postrelease in Queensland, Australia only rarely detected genetic polymorphisms (29). These studies suggest that the *w*Mel genome is quite stable in *Ae. aegypti*, which will presumably aid in the continuation of its antiviral properties in this host.

Plausibly, evolution of the *Ae. aegypti* genome could attenuate *w*Mel-mediated viral inhibition by adapting to the endosymbiont over time. Ford et al. selectively bred *w*Mel-infected mosquitoes that either established high or low levels of viral RNA after DENV infection. They found the low and high DENV levels were linked to genomic variation in *Ae. aegypti* (30). However, the mosquito phenotypes that were less resistant to viral infection were also less fit, suggesting they would be unlikely to be selected in the field.

The stability of the *Wolbachia-Ae. aegypti* association has been demonstrated in Queensland (19, 24) and Malaysia (31), where *w*Mel and *w*AlbB, respectively, were introgressed into the *Ae. aegypti* population. *Wolbachia* has remained at a high frequency in these mosquito populations for up to a decade, and has retained its antiviral properties (31, 32). Together, these studies indicate that the *Wolbachia-Ae. aegypti* relationship is unlikely to evolve rapidly in the field in a manner that quickly undermines the public health benefits of the *Wolbachia* introgression method.

In contrast to *Wolbachia* and *Ae. aegypti*, RNA viruses like DENV have much faster mutation rates. Viruses that accumulate mutations in the genome (variants) that can replicate in *Wolbachia-*carrying mosquitoes may be rapidly selected. These variants could be maintained in a *Wolbachia-Ae. aegypti* population provided they can replicate well within the human host. Thus, whether DENV will remain susceptible to the antiviral state created by *w*Mel and *w*AlbB infection in *Ae. aegypti* remains a key question to be addressed (33, 34). In this review, we examine the risk and potential mechanisms by which DENV resistance against *Wolbachia* might evolve and discuss how viral resistance to *Wolbachia* could be identified and managed operationally.

## SELECTION AND EMERGENCE OF *WOLBACHIA*-RESISTANT VIRUS IN MOSQUITOES

The urban transmission cycle sees DENV circulate between human and mosquito hosts. Mosquitoes become infected with DENV when the insect takes an infectious blood meal from a viremic person. Since *Wolbachia* resides within mosquitoes, selective pressure for the virus population to overcome *Wolbachia*’s antiviral properties will only be present in this part of the transmission cycle. While the emergence of viral resistance to antiviral therapeutics in humans is a relatively common phenomenon (35–37), selection pressures applied to DENV by *Wolbachia* are likely to differ in many ways. For instance, while antiviral drugs have a defined mode of action, the mode of action of *Wolbachia* appears to be broad and may be indirect (38). In addition, while therapeutics are administered at optimized concentrations and have well-defined pharmacological properties (39), *Wolbachia* abundance (density) cannot be easily controlled and varies between *Wolbachia* strains, individual mosquitoes, and host tissues (40–45). Control of the levels of DENV inhibition within specific *Ae. aegypti* tissues appears to be complex and is not just associated with *Wolbachia* density (40, 42, 46). In this section we postulate how *Wolbachia*-resistant DENV variants may emerge, based on our current understanding of DENV infection, dissemination, and transmission in mosquitoes.

### *w*Mel and *w*AlbB *Wolbachia* strains provide incomplete protection against DENV.

The *w*Mel and *w*AlbB *Wolbachia* strains used in field releases have been rigorously tested in laboratory studies to determine their impacts on DENV infection dynamics in *Ae. aegypti*. Broadly speaking, these strains provide partial protection against fulminant DENV infection compared to mosquitoes without *Wolbachia* (46). Most important to the effectiveness of these strains in the field is their ability to both reduce the proportion of *Ae. aegypti* with infectious DENV in their saliva (22, 23) and lengthen the extrinsic incubation period (time taken for mosquito saliva to become infectious following virus uptake in a blood meal), thus reducing the number of days in a mosquito’s life span in which it can infect people (22, 47). Nevertheless, *Wolbachia*-mediated viral inhibition is incomplete, such that a proportion of mosquitoes become infectious with DENV. For example, after feeding on blood from viremic dengue patients, infection was detected in the abdomen (53 to 61%) and saliva (6 to 12%) of *w*Mel-carrying mosquitoes (22, 23). Even at a population level, it has been estimated that introgression of *w*Mel would not eliminate DENV in high-transmission settings indefinitely (6). Also of note, DENV-1 is marginally less inhibited by *w*Mel than serotypes 2, 3, and 4 (22, 23). Plausibly, a smoldering pattern of DENV replication and transmission could provide the opportunity for *Wolbachia*-resistant viruses to emerge and be selected (48).

## PROCESS OF *WOLBACHIA*-RESISTANT VIRUS SELECTION

Within mosquito tissues, both *w*Mel-carrying and *w*Mel-free cells can be observed (42) and these cells are likely to possess different antiviral states. At the cellular level, Nainu et al. determined the antiviral effects of *w*Mel to be cell-autonomous (i.e., viral protection is limited to *Wolbachia*-infected cells) (49). JW18 *Drosophila* cells with *w*Mel were unable to protect *Wolbachia*-free JW18 cells from infection by *Drosophila* C Virus (DVC; *Dicistroviridae*, cripavirus) or Sindbis virus (SINV; *Togaviridae*, alphavirus) when cocultured in *trans*-wells separated by a porous membrane (49). Similarly, it seems that antiviral *Wolbachia* strains show a “superinfection exclusion-like phenotype,” whereby cells that have *Wolbachia* prevent productive viral infection (50, 51) and DENV and *Wolbachia* coinfected cells are rarely visualized in mosquito tissues and cell culture (50, 52). These studies suggest that *Wolbachia*-free cells within mosquito tissues that can support productive virus infection may be the site where *Wolbachia*-resistant virus types may emerge, followed by their selection in *Wolbachia*-carrying cells.

After ingesting a blood meal from a viremic person, DENV replicates in the *Ae. aegypti* midgut. The virus must then traverse the midgut barrier, enter the hemolymph, and infect other tissues, reaching the salivary glands after ∼7 days. Once the virus enters the mosquito’s saliva it can be passed to a new host when the next blood meal is taken.

**FIG 1:**
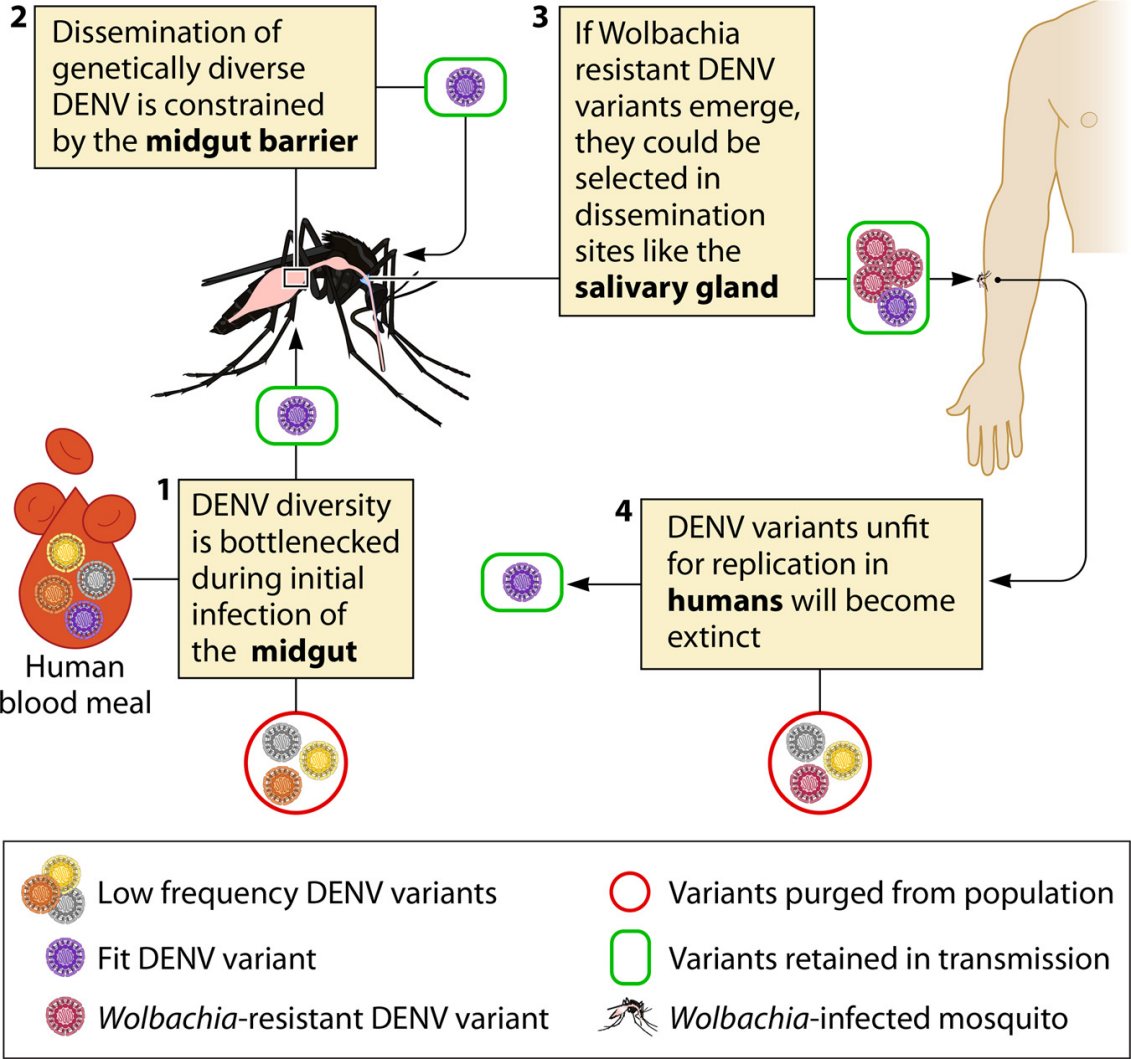
Potential pathways for selection and removal of a *Wolbachia*-resistant variant through the DENV transmission cycle. This schematic highlights the population bottlenecks and fitness trade-offs that could prevent *Wolbachia*-resistant DENV variants from persisting throughout the transmission cycle. The DENV population in a blood meal is genetically diverse, but only a small proportion of variants establish infection in the mosquito midgut (1) and are able to disseminate to distal tissues (2). Variants that are more resistant to the antiviral properties of *Wolbachia* may be selected, allowing the virus to replicate in *Wolbachia*-infected and -uninfected cells. These variants may possess a replicative advantage in disseminated sites of the mosquito with high *Wolbachia* density, such as the salivary gland (3). DENV variants that replicate efficiently in the mosquito might not always be infectious for humans (4), such that if a *Wolbachia*-resistant variant did infect a human, it may replicate poorly or be outcompeted by other variants that are better adapted for replication in humans.

In blood-fed mosquitoes, only a small number of infectious units are thought to seed infection in the mosquito midgut (53, 54). This reduction in virus population size, known as a population bottleneck, decreases the genetic diversity of the infective virus population (55, 56). This event may cause low-frequency *Wolbachia*-resistant DENV variants already present in the incoming blood meal to be filtered out (Fig. 1, step 1).

Replication of DENV in the midgut leads to the generation of viral variants because the virus lacks proofreading capacity. These variants may be unable to disseminate beyond the midgut if they have reduced competitive fitness (57) or are susceptible to immune mediators within the midgut and hemolymph (58) (Fig. 1, step 2).

If a fit *Wolbachia*-resistant DENV variant is generated in the midgut, this virus would possess a selective advantage over wild-type viruses in mosquitoes that carry *Wolbachia*. Selection may occur if the variant could similarly infect both *Wolbachia*-carrying and *Wolbachia*-free cells, or if a variant evolves to specifically target *Wolbachia*-free cells. Mechanistically, DENV could specifically target *Wolbachia*-free cells by adapting its affinity for viral entry receptors (59) to those that are differentially expressed between *Wolbachia*-free and -carrying cells. Lu et al. showed that *w*AlbB infection modulates the expression of DENV attachment factors dystroglycan and tubulin in Aag2 cells (60). Another study showed that expression of the cell surface insulin receptor is modulated by *w*Mel infection, reducing the susceptibility of mosquito cells to DENV and ZIKV (Zika virus) infection (61). While the insulin receptor is not a known entry receptor for DENV, this study illustrates that *Wolbachia* has the potential to modulate expression of cell membrane proteins and thereby alter the permissiveness of these cells to viral infection.

Preferential replication of *Wolbachia*-resistant DENV compared to wild-type virus would ultimately establish these fit viral variants in the salivary glands (Fig. 1, step 3).

Onward transmission of *Wolbachia*-resistant DENV variants would be limited if they are unfit in the human host (Fig. 1, step 4). This scenario would be considered a fitness trade-off, where fitness increases in one host (i.e., the mosquito) are counterbalanced by fitness losses in the second host (i.e., humans). Alternatively, if the variant can establish infection in the human host, onward transmission may occur.

### Evolutionary processes that impact maintenance of DENV variants.

Fitness trade-offs and population bottlenecks result in purifying selection, an evolutionary feature of DENV (62, 63). In purifying selection, synonymous mutations, which do not cause amino acid changes, are more likely to be maintained than nonsynonymous mutations. Purifying selection purges deleterious variants from the transmission cycle, many of which are caused by nonsynonymous mutations since these mutations can impact protein stability, function, and viral replication (64). Arguably, nonsynonymous mutations in viral proteins might be more successful than synonymous changes in escaping the selective pressures imposed by *Wolbachia*. But these variants must still support efficient viral replication.

While purifying selection may slow the emergence of *Wolbachia*-resistant variants, it may not eliminate them. *Wolbachi*a-resistant variants could accumulate over time, eventually becoming dominant in transmission cycles. Considering mosquito populations are large and their susceptibility to DENV infection can fluctuate, continued monitoring for virus evolution in *Wolbachia*-carrying mosquitoes will be important in regions where *Wolbachia*-carrying *Ae. aegypti* have been established.

Certainly, compared to antiviral resistance events described for viruses that circulate in a single host, the sequential evolutionary speed bumps that DENV populations encounter are likely to delay *Wolbachia*-resistant viruses from emerging in transmission cycles.

## *WOLBACHIA* IMPACTS ON THE SUBCELLULAR DENV INFECTIOUS CYCLE

*Wolbachia* is a complex organism thought to inhibit the infectious cycle of DENV within mosquito cells that carry the bacterium by numerous mechanisms (38). In the following section, we explore some of the proposed inhibitory mechanisms with the aim of speculating how DENV could evolve to bypass these on an intracellular level.

To determine the stage(s) of the DENV life cycle that are impacted by *Wolbachia*, the progression of viral infection has been tracked in insect cell lines artificially infected with *Wolbachia*. Consistently, it has been shown that viral replication is significantly reduced in mosquito or *Drosophila* cells when antiviral *Wolbachia* strains are present, and it is widely agreed that DENV (as well as other related flaviviruses and unrelated alphaviruses) are likely to be inhibited after virus entry, at an early stage in RNA replication, or perhaps at translation of the incoming viral RNA template (51, 65–67). It should be noted that, for practical reasons, many of the studies characterizing the impacts of *Wolbachia* at the cellular level have been performed using cell culture models. In whole mosquitoes, these effects may vary between tissues, as the virus encounters different *Wolbachia* densities, as well as cell type-specific effects during infection and dissemination.

### Overcoming *Wolbachia*-induced host effects that contribute to viral inhibition.

Both DENV and *Wolbachia* are known to alter their host environment. *Wolbachia* is present in the mosquito throughout its life cycle, and it is therefore likely that some of the *Wolbachia*-induced host changes interfere with essential stages of virus infection. Identifying how DENV is restricted will help us to determine how viral resistance may emerge against *Wolbachia*. Relevant host cell modifications induced by *Wolbachia* can be grouped into 3 main categories: host cell structural modifications, altered nutrient homeostasis, and induction of host immune/stress responses. Lindsey et al. provide a comprehensive review discussing the various ways *Wolbachia* may induce these changes and how they may impact on viral pathogens (38). While it is possible that DENV may adapt to overcome a specific antiviral factor that drives these modifications (either *Wolbachia*- or host-cell derived), we have kept our discussion broad, since it is not known which viral/antiviral factor interaction(s) is responsible for inhibiting DENV. Additionally, viral inhibition is probably induced by the collective contribution of several *Wolbachia*-induced host modifications (38). As such, several points in the DENV life cycle may be simultaneously under selective pressure when *Wolbachia* is present. While it is unlikely that a single mutation in the viral genome may allow complete viral resistance to emerge, it is possible that particular mutations may allow the virus to overcome one or some of these effects, reducing the overall impact of *Wolbachia* in inhibiting viral transmission. Here, we will focus on three subcellular modifications that are likely to be critical for *Wolbachia* to induce its antiviral effect, and consider whether viruses could adapt to overcome these pressures.

**(i) Altered lipid homeostasis.** *w*Mel and *w*AlbB infection of *Ae. aegypti* imparts minor costs on host fitness (41, 68, 69). Genomic studies of multiple *Wolbachia* strains show it must source a variety of amino acids and lipid complexes from its host to complement its own limited metabolic pathways (70). Several groups have examined the hypothesis that *Wolbachia* may alter the lipid profile of host cells, disrupting the requirements for productive DENV infection. Koh et al. (71) examined the lipid profile in whole *w*Mel-*Ae. aegypti* and DENV-infected *Wolbachia*-free *Ae. aegypti* (intrathoracically injected with DENV-3). They reported that DENV infection of mosquitoes induced a lipid profile distinct from mosquitoes carrying *w*Mel, suggesting that DENV-3 and *Wolbachia* are not in direct competition for lipids. In mosquitoes coinfected with DENV-3 and *w*Mel, they found that DENV modulation of host lipids dominates the changes normally induced by *Wolbachia*. However, it is important to note that intrathoracic DENV infections are known to overwhelm the effects of *Wolbachia* and may not represent the virus-*Wolbachia* relationship in a natural infection (68). Furthermore, analysis of the lipidome in whole mosquitoes may mask smaller, tissue-specific lipid changes induced by *Wolbachia*.

Manokaran et al. (72) also attempted to define the lipid changes that occur when *w*Mel and/or DENV is present in *Ae. aegypti*. Using the Aag2 *Ae. aegypti*-derived cell line, they identified acyl-carnitines (a class of lipids involved in energy production) as specifically upregulated by DENV and ZIKV, but downregulated in the presence of *w*Mel, including in *w*Mel-Aag2 following viral infection. This may suggest that *w*Mel and DENV are in fact competing for some lipids. The acyl-carnitine inhibitor etomoxir reduced DENV levels in *Ae. aegypti* without *w*Mel, supporting an *in vivo* role for this lipid. It is possible that this shift in acyl-carnitines occurs in only a subset of mosquito cell types, which could explain why it was not observed by Koh et al.

Other studies have also shown that supplementing or chemically modulating host lipid profiles in mosquito cell lines or *Drosophila* that carry various antiviral *Wolbachia* strains reduces the antiviral effectiveness of *Wolbachia* (73–75). This suggests that regardless of whether viruses are competing with *Wolbachia* for the same lipids, lipids are likely to contribute in some way to the antiviral state imposed by *Wolbachia*.

Flaviviruses are highly dependent on cholesterol and other lipids for virion entry and exit, and formation of modified endoplasmic reticulum (ER) membranes for viral RNA replication (vesicle packets) (76–78). DENV infection also causes an accumulation of acyl-carnitines in the midgut of *Ae. aegypti*, suggesting the virus may divert energy to better support its own replication (79).

**FIG 2:**
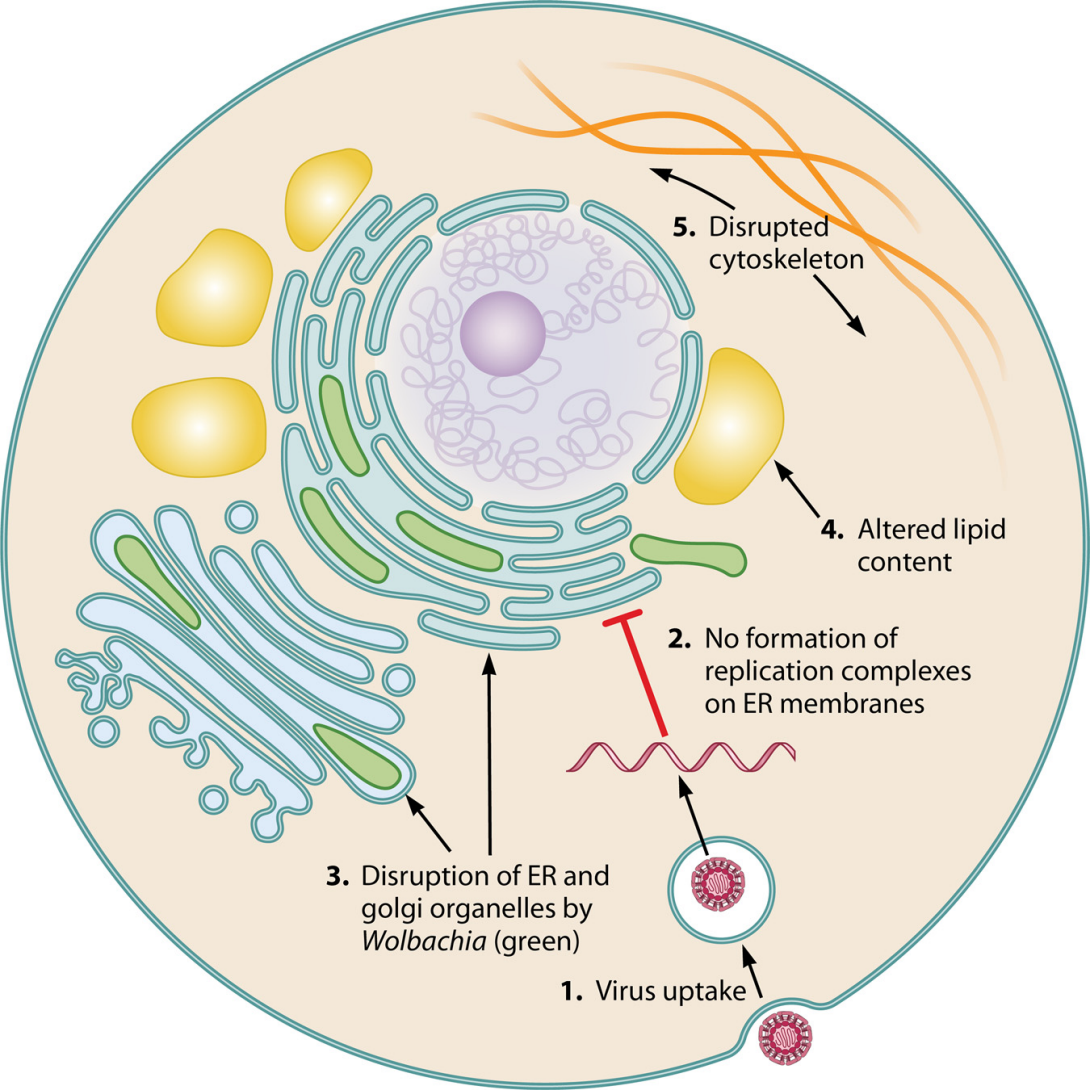
Proposed model of subcellular DENV restriction by *Wolbachia*. (1) Virus uptake occurs through clathrin-mediated endocytosis, and the viral genome is delivered following fusion of the viral and mature-endosomal membranes. (2) Replication of viral RNA (red) is restricted in *Wolbachia*-carrying cells and so is vesicle packet formation on ER membranes. This could be due to disturbance of ER and Golgi apparatus membranes due to (3) occupation/disruption by *Wolbachia* (green). (4) Altered cellular lipid content, e.g., increased cholesterol storage (yellow) or reduced acyl-carnitines, may restrict trafficking of membrane-bound vesicles and/or lower energy resources for virus production. Similarly, *Wolbachia*-induced alterations of the host cell cytoskeleton (5) may interfere with trafficking of endosomes and/or ER and Golgi vesicles required for movement of incoming virions and the maturation of daughter virions.

Perhaps *Wolbachia*-modulated lipid levels change the cholesterol content of cellular membranes to impair intracellular trafficking or formation of membrane-associated replication complexes, or reduce energy availability for DENV replication (Fig. 2). Further work is needed to determine if these hypotheses hold true and whether DENV can adapt to overcome these cellular changes.

**(ii) Disruption of intracellular membranes.** Studies examining *w*Mel in a *Drosophila*-derived cell line have shown that *Wolbachia* is intimately associated with host cell membranes. *w*Mel is contained within and around ER and Golgi-derived vesicles, causing regions of these organelles to swell (80–82). Given that specific remodeling of these organelles is required by DENV for replication and maturation, it seems likely that their disruption by *Wolbachia* could impair the establishment of viral infection. Work from Bhattacharya et al. has shown that the small amount of virus produced from insect cells carrying the *w*Mel strain of *Wolbachia* has reduced infectivity and/or replication capacity in mammalian cells (51, 67). This would be consistent with disrupted ER/Golgi structures, which are strictly required for viral RNA replication and the production and maturation of envelope proteins for flaviviruses (Fig. 2). In this scenario, perhaps disruption of viral RNA replication events could lead to the production of defective interfering viral particles (viruses that contain substantial deletions in their genomes) and/or perturbed ER/Golgi organelles may not allow the correct maturation and processing of viral envelope proteins, i.e., events which could reduce the infectivity of any viral particles produced.

Notably, while alphaviruses replicate and form virions in quite distinct regions of the cell compared to flaviviruses, alphaviruses are still dependent on their replication complexes forming in association with ER membranes, and trafficking and maturation of their envelope proteins through the ER and Golgi secretory pathway (83). Thus, disruption of these organelles could potentially similarly impact the two virus families.

It is yet to be determined whether *w*Mel or other antiviral *Wolbachia* strains similarly occupy these organelles *in vivo* in *Ae. aegypti*, but it is certainly a compelling hypothesis for a mechanism that may contribute to *Wolbachia*’s antiviral activity.

If *Wolbachia* is colonizing regions of the ER and Golgi, preventing typical establishment of DENV replication complexes and virus-specific remodeling events at these organelles, then perhaps the virus could adapt to replicate in regions unaffected by *Wolbachia*, or else could adapt to bud from the plasma membrane like alphaviruses. Given the intricate association of DENV with these organelles, from viral replication to virion formation and budding, it seems that these adjustments would take an enormous number of compensatory mutations arising across interacting viral proteins, before functional virus would emerge.

**(iii) Changes to the host cell cytoskeleton.** Other studies in *Drosophila* have revealed that *Wolbachia* utilizes microtubules and actin to support its localization, particularly in the *Drosophila* oocyte. This may allow the endosymbiont to persist throughout *Drosophila* development and to pass from generation to generation (84–86). Furthermore, *Wolbachia* has been shown to secrete the actin bundler protein WalE1. Overexpression of WalE1 in transgenic flies leads to an increase in *Wolbachia* titer, suggesting *Wolbachia* may manipulate actin to modulate its own replication (87). For DENV, each aspect of the virus life cycle, including entry, intracellular transport, replication, and egress is intimately tied to the host cell cytoskeleton. For example, DENV entry is dependent on actin filament integrity (88, 89), while organelle remodeling and formation of vesicle packets are associated with changes in the cytoskeleton structure, including reorganization of the intermediate filament vimentin—critical for DENV replication (90, 91). A situation where *Wolbachia* modulates the cytoskeleton to disrupt DENV trafficking into cells and/or formation of vesicle packets would be consistent with *Wolbachia* restricting DENV at a stage prior to RNA replication (Fig. 2) (51, 65, 66).

If *Wolbachia* disrupts DENV entry via endocytosis, could DENV entry adapt to occur in a pH-independent manner, at the cell surface? There have been reports that alphaviruses, including SINV, may be able to enter both by receptor-mediated endocytosis and at the plasma membrane (92, 93). This would require mutations to accumulate in the viral envelope protein that allow fusion activation (conformational changes in the envelope proteins that drive the merging of viral and host cell membranes) to occur at a neutral and acidic pH.

In fact, pH-independent entry has been described in laboratory experiments for flaviviruses and related hepaciviruses. Endosomal fusion activation events for these viruses are crucially controlled by specific histidine residues within the viral fusion protein (94–96). Boo et al. demonstrated that substitution of histidine with positively charged arginine enhanced entry of hepatitis C virus (hepacivirus) at neutral pH (94).

## VIRUS FAMILIES THAT ARE RESISTANT TO *WOLBACHIA*

Perhaps another way we can consider how viral resistance may arise against *Wolbachia* is to examine the life cycle of viruses that are not inhibited by this endosymbiont. There are several reports that negative-sense RNA viruses, including bunyaviruses, are not inhibited by *Wolbachia*. The insect-specific virus Phasi Charoen-like virus (*Bunyaviridae*) can replicate effectively as both a persistent infection and following an acute challenge in the *Ae. aegypti*-derived cell line Aag2 coinfected with *w*Mel or *w*MelPop *Wolbachia* strains (97, 98).

Bunyaviruses typically have three negative-sense RNA segments that are bound to multiple copies of the viral polymerase (L) and nucleoprotein (N), encased in a lipid bilayer. Similar to flavi- and alphaviruses, bunyaviruses are internalized via clathrin-mediated endocytosis, and transcription and translation are closely coupled, occurring in association with the rough ER (see reference 99 for a review on bunyavirus replication). However, the replication strategy for these viruses differs substantially to flavi- and alphaviruses, since the incoming viral RNA must be transcribed to a positive-sense RNA (generating either an mRNA for translation or a positive-sense replicative intermediate), with the replicative intermediate copied again to generate the negative-sense progeny viral RNAs. Interestingly, these progeny viral RNAs may associate with newly formed L and N proteins in a structure called the viral tube before budding through the Golgi, where it collects its membrane and envelope proteins (100). Perhaps this distinct RNA replication and assembly strategy, whereby shorter viral RNAs are protected by L and N proteins at each stage, enables bunyaviruses to persist in the presence of *Wolbachia*.

## INVESTIGATING EVOLUTION OF *WOLBACHIA*-RESISTANT VIRUSES

Further studies into the evolution of DENV in the presence of *Wolbachia* may direct us toward the mechanisms that underlie viral inhibition by indicating the regions of the genome that are under selective pressure. This in turn may allow us to predict the likelihood of these mutations arising in the field. To do this, we can use a laboratory setting to push conditions to favor viral sequence diversity. By continually passaging DENV in an invertebrate host with *Wolbachia* (whole insects or cell culture), we can remove the purifying selection usually associated with host alternation in order to broaden the repertoire of viral sequences being maintained over time.

Such studies have been reported by two groups. One study passaged the RNA virus DCV through whole D. melanogaster with a native *w*MelCS infection (a strain closely related to *w*Mel) over 10 passages (101). The other study passaged DENV-3 ten times in *Ae. aegypti*-derived Aag2 cells artificially infected with *w*Mel (102). In both studies, the viruses replicated over time when consistently challenged by *Wolbachia*. However, these viruses grew to substantially lower titers and had no replicative advantage over those passaged in *Wolbachia*-free cells. Notably, no studies have yet examined viral passaging in the presence of *w*AlbB.

While these are very artificial evolutionary experiments, they show that, in the laboratory, RNA viruses do not develop fit viral variants with resistance to *w*Mel in a short time frame.

## DETECTION AND MANAGEMENT OF *WOLBACHIA*-RESISTANT DENV

If a fit DENV variant that is able to replicate in *Wolbachia*-carrying mosquitoes were to establish itself in a transmission cycle, how would it be identified and how would we mitigate the impact? In regions such as Yogyakarta, Indonesia, where local transmission of DENV has ceased in areas where *Wolbachia* has been introgressed into *Ae. aegypti* populations (26), viral resistance could be suspected if persistent local DENV transmission chains were reported in areas of *Wolbachia* establishment. Since loss of *Wolbachia*-mediated virus inhibition could occur due to changes in the virus, *Wolbachia*, or the *Ae. aegypti* host, it would be essential to first determine the underlying cause(s) of the transmission events.

Before assuming that a virus has evolved resistance to *Wolbachia*, it would be prudent to ensure *Wolbachia* has not been substantially reduced in density or frequency within a mosquito population, e.g., due to exposure to very high temperatures (103). It would also be important to rule out adaptation of the mosquito host or *Wolbachia*, which may allow the mosquito population to become permissive to DENV infection (30). This could be done by challenging wild-caught *Wolbachia-*carrying *Ae. aegypti* with a blood-meal spiked with laboratory viruses previously shown to be inhibited by that *Wolbachia* strain.

To determine if viral resistance is the underlying cause of the transmission events, laboratory *Wolbachia*-carrying *Ae. aegypti* colonies could be infected with circulating virus isolates from the region. Measuring the replication/transmission of these viruses in laboratory-reared mosquitoes, alongside previously published *Wolbachia*-sensitive laboratory viral strains, would determine if the DENV genotypes circulating in the community were better able to overcome the inhibitory effects of *Wolbachia*. Sequencing of the circulating DENV isolates from both human and mosquito hosts over the course of an outbreak and comparison with recent historical isolates may provide insight into the genetic changes that may have led to viral resistance.

Viral resistance against an introgressed *Wolbachia* strain could be managed using various strategies. One option is to not alter the existing mosquito population, as it is unlikely that *Wolbachia*-carrying mosquitoes would be more susceptible to DENV than wild-type mosquitoes. Initially, it is likely that only one DENV genotype would be resistant to the antiviral properties of *w*Mel or *w*AlbB, and *Wolbachia* may still protect against all other genotypes/serotypes. Over time, the resistant genotype would likely become dominant, and in this scenario supplementary interventions may be of benefit. Releases of mosquitoes that carry a reproductively incompatible *Wolbachia* strain could be performed to remove an existing strain or to replace it as long as viral resistance does not extend to all *Wolbachia* strains. Management of viral resistance could also be achieved through the use of complementary interventions, such as vaccines or vector control strategies that are based on gene drive and/or population suppression. While many of these complementary methods are still undergoing development and evaluation, initial reports indicate they show potential for future implementation (14, 104, 105).

## CONCLUDING REMARKS

With a body of evidence now demonstrating that *Wolbachia-Ae. aegypti* introgression methods can substantially reduce the burden of dengue in areas of endemicity, it is expected that application of this technology will undergo a major expansion in coming years (17, 19, 24, 26). The intention is that this will lead to long-term control or local elimination of human-pathogenic arboviruses. Achieving long-term suppression in the field would be dependent upon the evolutionary stability of the *Wolbachia*, *Ae. aegypti*, and DENV tripartite interaction. *Wolbachia* and *Ae. aegypti* evolve slowly compared to DENV, and *Wolbachi*a-carrying mosquitoes collected years after release have so far retained their antiviral profile. Yet the rapid mutation rate of RNA viruses suggests it is inevitable that viruses like DENV will eventually adapt to *Wolbachia*’s selective pressure and become resistant to the intervention. The question is, how long will this take?

There is no precedent for an antiviral intervention like *Wolbachia*, and we cannot be certain how viruses will adapt upon continued exposure to this endosymbiont. In the field, DENV will repeatedly face the selective pressures imposed by *Wolbachia*, but the genetic diversity generated and maintained by the virus will be limited by the need for the virus to infect a range of mosquito tissues, while also maintaining competence in the human host. In addition, since the mode of action of *Wolbachia* appears broad, it is most likely that multiple mutations across the viral genome will be necessary to allow the virus to adapt to this unique cellular landscape.

While we have focused on factors that may affect the development of viral resistance to *Wolbachia*-introgression methods, these considerations are also highly relevant to any gene drive/replacement technology where the virus and host will coexist in a long-term evolutionary relationship. Finally, as *Wolbachia*-based biocontrol methods increase in scope and longevity, monitoring for the emergence of viral resistance to *Wolbachia* should remain a critical component of these programs.
