# Correction for Edenborough et al., “Using *Wolbachia* to Eliminate Dengue: Will the Virus Fight Back?”

**DOI:** 10.1128/JVI.00953-21

**Published:** 2021-08-25

**Authors:** Kathryn M. Edenborough, Heather A. Flores, Cameron P. Simmons, Johanna E. Fraser

**Affiliations:** a Institute of Vector-Borne Disease, Monash University, Clayton, Victoria, Australia; b World Mosquito Program, Institute of Vector-Borne Disease, Monash University, Clayton, Victoria, Australia; c Oxford University Clinical Research Unit, Ho Chi Minh City, Vietnam; d Nuffield Department of Medicine, University of Oxford, Oxford, United Kingdom; e Department of Microbiology, Monash Biomedicine Discovery Institute, Monash University, Clayton, Victoria, Australia

## AUTHOR CORRECTION

Volume 95, no. 13, e02203-20, 2021, https://doi.org/10.1128/JVI.02203-20. The article was originally published on 10 June 2021 with a standard copyright line (“© 2021 American Society for Microbiology. All Rights Reserved.”). We elected to pay for open access for the article after publication, necessitating replacement of the original copyright line with “© 2021 Edenborough et al. This is an open-access article distributed under the terms of the Creative Commons Attribution 4.0 International license.” This change was made to the online version of the article on 2 July 2021.

